# Variability of Myocardial Strain During Isometric Exercise in Subjects With and Without Heart Failure

**DOI:** 10.3389/fcvm.2020.00111

**Published:** 2020-06-30

**Authors:** Moritz Blum, Djawid Hashemi, Laura Astrid Motzkus, Marthe Neye, Aleksandar Dordevic, Victoria Zieschang, Seyedeh Mahsa Zamani, Tomas Lapinskas, Kilian Runte, Marcus Kelm, Titus Kühne, Elvis Tahirovic, Frank Edelmann, Burkert Pieske, Hans-Dirk Düngen, Sebastian Kelle

**Affiliations:** ^1^Department of Internal Medicine/Cardiology, Charité–Universitätsmedizin Berlin, Berlin, Germany; ^2^DZHK (German Center for Cardiovascular Research), Berlin, Germany; ^3^Department of Internal Medicine/Cardiology, German Heart Center Berlin, Berlin, Germany; ^4^Department of Cardiology, Medical Academy, Lithuanian University of Health Sciences, Kaunas, Lithuania; ^5^Department of Congenital Heart Disease, German Heart Center Berlin, Berlin, Germany; ^6^Institute for Imaging Science and Computational Modelling in Cardiovascular Medicine, Charité–Universitätsmedizin Berlin, Berlin, Germany

**Keywords:** heart failure, cardiac magnetic resonance imaging, strain, fast SENC, isometric handgrip

## Abstract

**Background:** Fast strain-encoded cardiac magnetic resonance imaging (cMRI, fast-SENC) is a novel technology potentially improving characterization of heart failure (HF) patients by quantifying cardiac strain. We sought to describe the impact of isometric handgrip exercise (HG) on cardiac strain assessed by fast-SENC in HF patients and controls.

**Methods:** Patients with stable HF and controls were examined using cMRI at rest and during HG. Left ventricular (LV) global longitudinal strain (GLS) and global circumferential (GCS) were derived from image analysis software using fast-SENC. Strain change < -0.5 and > +0.5 was classified as increase and decrease, respectively.

**Results:** The study population comprised 72 subjects, including HF with reduced, mid-range and preserved ejection fraction and controls (HFrEF *n* = 18 HFmrEF *n* = 18, HFpEF *n* = 17, controls: *n* = 19). In controls, LV GLS remained stable in 36.8%, increased in 36.8% and decreased in 26.3% of subjects during HG. In HF subgroups, similar patterns of LV GLS response were observed (HFpEF: stable 41.2%, increase 35.3%, decrease: 23.5%; HFmrEF: stable 50.0%, increase 16.7%, decrease: 33.3%; HFrEF: stable 33.3%, increase 22.2%, decrease: 44.4%, *p* = 0.668). Mean change between LV GLS at rest and during HG ranged close to zero with broad standard deviation in all subgroups and was not significantly different between subgroups (+1.2 ± 5.4%, −0.6 ± 8.3%, −1.7 ± 10.7%, and −3.1 ± 19.4%, *p* = 0.746 in controls, HFpEF, HFmrEF and HFrEF, respectively). However, the absolute value of LV GLS change—irrespective of increase or decrease—was significantly different between subgroups with 4.4 ± 3.2% in controls, 5.9 ± 5.7% in HFpEF, 6.8 ± 8.3% in HFmrEF and 14.1 ± 13.3% in HFrEF (*p* = 0.005). The absolute value of LV GLS change significantly correlated with resting LVEF, NTproBNP and Minnesota Living with Heart Failure questionnaire scores.

**Conclusion:** The response to isometric exercise in LV GLS is heterogeneous in all HF subgroups and in controls. The absolute value of LV GLS change during HG exercise is elevated in HF patients and associated with measures of HF severity. The diagnostic utility of fast-SENC strain assessment in conjunction with HG appears to be limited.

**Trial Registration:** URL: https://www.drks.de; Unique Identifier: DRKS00015615.

## Introduction

Heart Failure (HF) remains a significant burden for patients and health systems worldwide and, with high mortality despite optimal therapy, refinement of therapeutic and diagnostic strategies is needed (1). Different phenotypes in HF—namely HF with preserved, mid-range and reduced ejection fraction (HFpEF, HFmrEF, and HFrEF, respectively) (2)—respond differentially to medical therapy (3–6). Thus, accurate diagnosis and stratification of HF patients is of paramount importance.

Cardiac strain is an emerging diagnostic target in cardiac imaging, describing myocardial deformation throughout the cardiac cycle in three dimensions (7). Global longitudinal strain (GLS) and global circumferential strain (GCS) have been shown to be more sensitive in detecting myocardial dysfunctions than left ventricular (LV) ejection fraction (EF) and therefore promise earlier diagnosis and initiation of treatment (8, 9). Also, strain could facilitate accurate stratification of and consecutively appropriate therapy for HF patients (10). Cardiac magnetic resonance imaging (cMRI) represents the gold standard for cardiac imaging, especially for measuring volumes (2). Among other methods to quantify myocardial strain in cMRI, such as myocardial tagging, displacement encoding with stimulated echoes (DENSE) and feature tracking (FT) (11–13), fast strain-encoded cMRI (fast-SENC) is a relative novel approach which allows for reproducible and fast strain measurement (14, 15).

Physical stress testing can unmask myocardial dysfunction in early stages of HF—especially in HFpEF (16). For stress testing during cMRI, isometric handgrip exercise (HG) represents an accessible and reliable tool, potentially avoiding motion artifacts associated with dynamic exercise. The combination of strain analysis and HG has successfully been employed for detection of ischemia and could provide a new diagnostic approach for HF (17). HG represents an acute increase in afterload which physiologically is met by an elevation in HR and an increase of cardiac output. In patients with a poor cardiac reserve a rise in the left ventricular end-diastolic pressure can be expected (18). Also an effect on myocardial performance indices such as global strain might therefore be conceivable. Therefore, in this study we sought to characterize the impact of HG stress testing on cardiac strain assessed by fast-SENC, in HF patients and healthy controls.

## Methods

### Study Population

The *Analysis of parameters of external and internal cardiac power, output and aortal compliance using cardiac MRI in patients with HF study* (EMPATHY-HF) was an investigator-initiated, prospective, cross-sectional study (German Clinical Trials Register ID: DRKS00015615). The study was performed in compliance with the Declaration of Helsinki and the study protocol was approved by the local institutional review board (Ethikausschuss 4 am Campus Benjamin Franklin, Charité Universitätsmedizin Berlin). All patients provided written informed consent before entering the study. A dedicated analysis of specific resting cMRI parameters derived from this study population has been published previously (19).

We included patients with stable chronic HF. Inclusion criteria are described in detail elsewhere (20). In brief, dyspnea NYHA class II or more and NTproBNP ≥ 220 ng/l were required for all HF patients, while specific imaging requirements applied for HFpEF (LV EF ≥ 50%, E/e' ≥ 13 or left atrial volume index >34 mL/m^2^ or LV septum thickness >12 mm), HFmrEF (LV EF 40–49%) and HFrEF (LV EF ≤ 40%), as per European Society of Cardiology guidelines (2). All patients had to receive medical therapy as recommended in current guidelines. Additionally, we included controls without HF.

Exclusion criteria included atrial fibrillation (AF), high-grade valvular disease or a history of valve replacement, and cMRI contraindications such as implanted cardioverter-defibrillator (ICD) or pacemaker, BMI >38 kg/m^2^ as well as a history of adverse contrast-medium reaction.

### Study Procedures

All subjects underwent comprehensive clinical work-up including physical examination laboratory evaluation, ECG, 6-min walk test and quality of life assessment using the Minnesota Living with Heart Failure Questionnaire (MLHFQ). Medical history, current diagnoses and medication were extracted from electronic health records.

**Figure 1:**
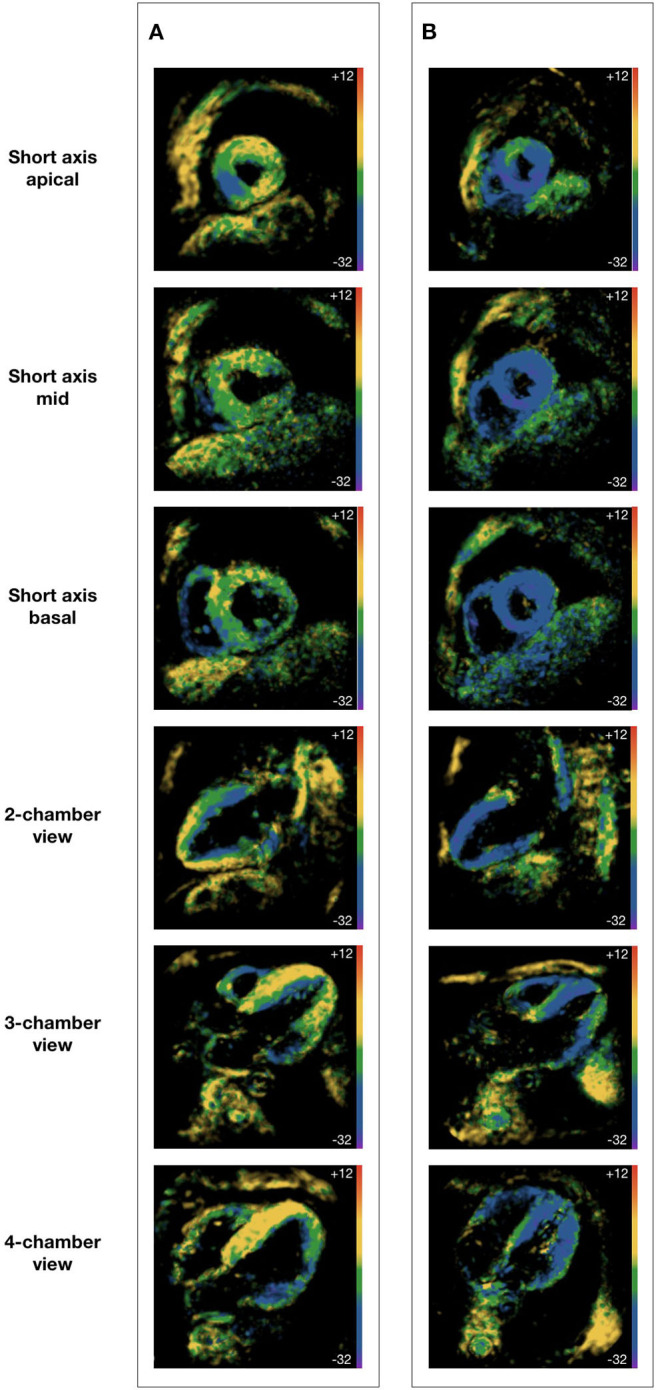
Fast strain-encoded cardiac magnetic resonance imaging. **(A)** 62 year-old male with heart failure with reduced ejection fraction; **(B)** 62 year-old male without heart disease; all subjects were at physical rest during image acquisition; all images were acquired at end-systole; global longitudinal strain is derived from short axis views at apical, mid and basal level; global circumferential strain is derived from two-, three-, and for-chamber views.

CMRI was performed using a clinical 1.5 Tesla MRI scanner (Achieva, Philips Healthcare, Best, The Netherlands) with a cardiac five-element phased array coil. Cine images were acquired using a retrospectively gated cine-cMRI in cardiac short-axis, vertical long-axis and horizontal long-axis orientations using a steady-state free precession sequence at rest. Fast-SENC was acquired at rest and during HG in real-time free breathing technique, as described previously (14). In brief, this SENC method generates temporary markers within the myocardium based on the unique MRI properties of tissue. The deformation of the myocardium during the cardiac phases changes the density of the markers, which when captured using an MRI spiral acquisition produces a cine sequence of SENC images (Figure 1). Three short-axis planes (apical, mid, and basal level) as well as two-, three- and for-chamber planes were assessed.

After 15 min of supine rest, resting blood pressure and heart rate were obtained, followed by resting cMRI sequences. For HG exercise testing, a MRI-safe hand dynamometer was used (Stoelting, Wood Dale, Illinois). After determination of maximum voluntary contraction using the dominant hand, subjects were instructed to sustain 30% of maximum voluntary contraction for ~3 min, avoiding Valsalva maneuver by continued breathing. Continuing HG exercise, blood pressure, heart rate and stress cMRI sequences were recorded under stress conditions.

### Image Analysis

Image analysis was performed using the software Medis® Suite 3.1.16.8 (Medis medical imaging systems, Leiden, The Netherlands) for left ventricular volume, mass and function measurements and the software MyoStrain 5.0 (Myocardial Solutions, Inc., Morrisville, North Carolina, USA) for fast-SENC strain measurements.

Trained operators manually traced endocardial and epicardial borders at end-systole and end-diastole. For quantitative assessment of global longitudinal strain (GLS), 3 short-axis planes (apical, mid and basal level) were analyzed using MyoStrain. Strain was calculated for each myocardial segment and then averaged. For quantitative global circumferential strain (GCS) assessment, three long-axis planes (two-, three-, and for-chamber view) were analyzed using the same software. Strain was calculated for each myocardial segment and then averaged. Myocardial shortening during systole translates to negative strain values. When communicating comparisons of strain values (e.g., strain decrease or increase) we will refer to the absolute value of strain, as recommended elsewhere (7).

We classified strain response to HG as *stable, increase* or *decrease*. In a recent study, our group investigated intra-observer reliability for LV GLS assessment employing fast-SENC in very similar cohort of healthy subjects and HF patients and found limits of agreement of −0.6 and +0.5 (15). Based on this observation, we decided that in order to classify as *increase* or *decrease*, LV GLS change must exceed < -0.5 or >+0.5, respectively. Accordingly, LV GLS change between ≥-0.5 and ≤ +0.5 was classified as *stable*.

### Statistical Analysis

Continuous variables are reported as mean (standard deviation), while categorical variables are reported as percentage. After testing for non-normality in distribution of continuous variables using the Shapiro-Wilk test, independent sample *t*-test, paired sample *t*-test and analysis of variance (ANOVA) for continuous response variables and Chi-square test for categorical response variables were used, as appropriate. For post-hoc analysis of intergroup differences in ANOVA we used Tukey's test. Pearson's coefficients were used to assess correlations between two continuous variables. For logarithmic transformations, the natural logarithm of variables of interest was utilized. Two-sided *p* < 0.05 were considered statistically significant. Sample size was chosen pragmatically based on similar previous studies and available research capacities (15, 21–23). Power calculation demonstrated that with the achieved sample size of *n* = 18 per subgroup and a standard deviation in LV GLS percentage change of ±12 overall, we were able to detect a subgroup difference of ±5 in LV GLS percentage change in ANOVA at a significance level of 0.05 yielding a statistical power of 0.83 (24).

Statistical analysis was performed using R version 3.5.1 (2018-07-02) (R Foundation for Statistical Computing, Vienna, Austria).

## Results

### Study Population

The final analysis comprised 72 subjects, 18 HFrEF patients, 18 HFmrEF patients, 17 HFpEF patients and 19 controls.

Baseline characteristics varied widely between subgroups (Table 1). HFpEF patients were the oldest, most likely to be female and had the highest prevalence of hypertension and diabetes mellitus. HFmrEF patients had the highest prevalence of coronary artery disease but had the least severe dyspnea symptoms according to New York Heart Association (NYHA) classification. HFrEF patients were most likely to be men, had the highest BMI the most smoking pack years on average.

**Table 1:** Baseline characteristics.

	**Controls *n* = 19**	**HFpEF *n* = 17**	**HFmrEF *n* = 18**	**HFrEF *n* = 18**	***p*-value**
Female Sex–no. (%)	9 (47.4)	8 (47.1)	6 (33.3)	3 (16.7)	0.176
Age–years	61.5 ± 8.1	77.9 ± 8.0	67.9 ± 9.2	65.4 ± 10.5	<0.001
BMI–kg/m^2^	25.1 ± 3.2	27.6 ± 3.8	27.3 ± 4.6	28.1 ± 3.8	0.104
CAD–no. (%)	0 (0.0)	11 (64.7)	15 (83.3)	13 (72.2)	<0.001
Hypertension–no. (%)	7 (36.8)	15 (88.2)	14 (77.8)	15 (83.3)	0.002
Previous MI–no. (%)	0 (0.0)	7 (41.2)	14 (77.8)	8 (44.4)	<0.001
Previous PCI–no. (%)	0 (0.0)	9 (52.9)	14 (77.8)	12 (66.7)	<0.001
Diabetes mellitus–no. (%)	2 (10.5)	5 (29.4)	3 (16.7)	5 (27.8)	0.441
LBBB on ECG–no. (%)	0 (0.0)	0 (0.0)	1 (5.6)	2 (11.1)	0.281
Ever Smoked–no. (%)	4 (21.1)	8 (47.1)	15 (83.3)	13 (72.2)	0.001
Packyears–years	2.0 ± 4.3	3.9 ± 6.8	29.8 ± 32.1	34.7 ± 52.7	0.003
NYHA Class II–no. (%)	0 (0.0)	10 (58.8)	15 (83.3)	15(72.2)	<0.001
III–no. (%)	0 (0.0)	7 (41.2)	3 (16.7)	3 (27.8)	
Leg Edema–no. (%)	3 (15.8)	12 (70.1)	14 (77.8)	12 (66.7)	<0.001
6 min walk distance–m	523.0 ± 118.6	344.4 ± 118.3	411.7 ± 86.0	417.4 ± 122.8	<0.001
MLHFQ QOL Score	4.7 ± 5.7	31.0 ± 23.1	25.2 ± 21.0	30.6 ± 25.8	<0.001
**CONCOMITANT MEDICATION**					
Beta-Blocker–no. (%)	6 (31.6)	11 (64.7)	14 (77.8)	17 (94.4)	<0.001
ACE-Inhibitor - no. (%)	2 (10.5)	3 (17.6)	6 (33.3)	10 (55.6)	0.015
ARB–no. (%)	4 (21.1)	12 (70.6)	7 (38.9)	8 (44.4)	0.027
MRA–no. (%)	0 (0.0)	3 (17.6)	4 (22.2)	11 (61.1)	<0.001
ARNI–no. (%)	0 (0.0)	1 (5.9)	0 (0.0)	4 (22.2)	0.026
Statin–no. (%)	2 (10.5)	9 (52.9)	15 (83.3)	11 (61.1)	<0.001
Loop Diuretic–no. (%)	0 (0.0)	3 (17.6)	6 (33.3)	7 (38.9)	0.02
HCT–no. (%)	4 (21.1)	4 (23.5)	2 (11.1)	1 (5.6)	0.401
**LABORATORY**					
Hb–g/dl	14.0 ± 1.1	13.0 ± 1.3	13.7 ± 1.1	14.9 ± 1.2	<0.001
RBC–/pl	4.7 ± 0.4	4.4 ± 0.5	4.5 ± 0.5	4.9 ± 0.5	0.007
WBC–/nl	6.1 ± 1.5	7.2 ± 2.4	8.5 ± 2.4	8.3 ± 2.3	0.003
Platelets–/nl	263.4 ± 65.9	265.8 ± 74.9	266.9 ± 74.0	209.7 ± 48.2	0.03
Hematocrit	0.40 ± 0.03	0.38 ± 0.03	0.41 ± 0.03	0.43 ± 0.04	0.001
Cholesterol–mg/dl	203.5 ± 33.5	172.5 ± 35.4	154.2 ± 44.3	156.1 ± 37.3	<0.001
LDL–mg/dl	133.0 ± 39.4	106.8 ± 29.5	92.2 ± 39.2	87.8 ± 30.4	0.001
HDL–mg/dl	66.5 ± 25.3	52.6 ± 12.8	49.4 ± 14.7	51.6 ± 18.3	0.029
Triglycerides–mg/dl	130.4 ± 79.6	129.7 ± 50.2	137.9 ± 81.1	173.3 ± 153.1	0.508
HbA1c –%	5.4 ± 0.5	5.9 ± 0.8	5.9 ± 0.7	5.8 ± 0.82	0.215
NTproBNP–ng/l	88.7 ± 61.1	459.1 ± 470.3	543.7 ± 385.5	2413.1 ± 3417.3	0.001
logNTproBNP–ng/l	4.26 ± 0.73	5.72 ± 1.13	6.06 ± 0.73	7.01 ± 1.20	<0.001
Hs TroponinT– ng/l	7.1 ± 3.4	19.9 ± 18.2	18.2 ± 19.67	19.4 ± 12.4	0.029
CRP–mg/l	1.3 ± 1.4	2.9 ± 2.7	3.1 ± 4.2	1.1 ± 0.7	0.029
**CARDIAC MRI PARAMETERS**					
LVEF –%	61.6 ± 5.4	61.6 ± 6.1	45.1 ± 2.7	33.5 ± 4.9	<0.001
LV EDV–ml	148.0 ± 34.5	130.3 ± 35.5	175.9 ± 28.8	261.8 ± 59.4	<0.001
LV ESV–ml	56.1 ± 18.4	50.9 ± 18.5	96.8 ± 17.7	175.6 ± 48.5	<0.001
LV SV–ml	90.1 ± 17.4	79.3 ± 20.1	79.1 ± 12.7	86.2 ± 16.6	0.144

*ARB, angiotensin receptor blocker; ARNI, angiotensin receptor blocker–neprilysin inhibitor; BP, blood pressure EDV, end-diastolic volume; EF, ejection fraction; ECG, electrocardiogram; ESV, end-systolic volume; GCS, global circumferential strain; GLS, global longitudinal strain; HF, heart failure; HFpEF, HF with preserved EF; HFmrEF, HF with mid-range EF; HFrEF, HF with reduced EF; LBBB, left bundle branch block; MI, myocardial infarction; MLHFQ, Minnesota living with heart failure questionnaire; MRA, mineralocorticoid receptor antagonist, PCI, percutaneous coronary intervention; QOL, quality of life; RBC, red blood cells; WBC, white blood cells*.

On laboratory examination, HFpEF patients had lowest levels of N-terminal pro-brain natriuretic peptide (NTproBNP), hemoglobin and red blood cells, but the highest levels of low-density-lipoprotein cholesterol, high-sensitivity Troponin T, high-sensitivity C-reactive protein, compared to other HF patients.

Almost all HFrEF patients received beta blockers (BB), and a majority also received angiotensin-converting enzyme inhibitors (ACEI) and mineralocorticoid antagonists (MRA). 22% of HFrEF patients received an angiotensin receptor blocker / neprilysin inhibitor (ARNI). HFmrEF patients were less likely to receive BB, ACEI or ARB and MRA compared to HFrEF patients. Among HFpEF patients, a majority received BB and either ACEI or ARB, 17.6% received MRA and one patient received off-label ARNI.

### Hemodynamic Features at Rest and During Exercise

**Figure 2:**
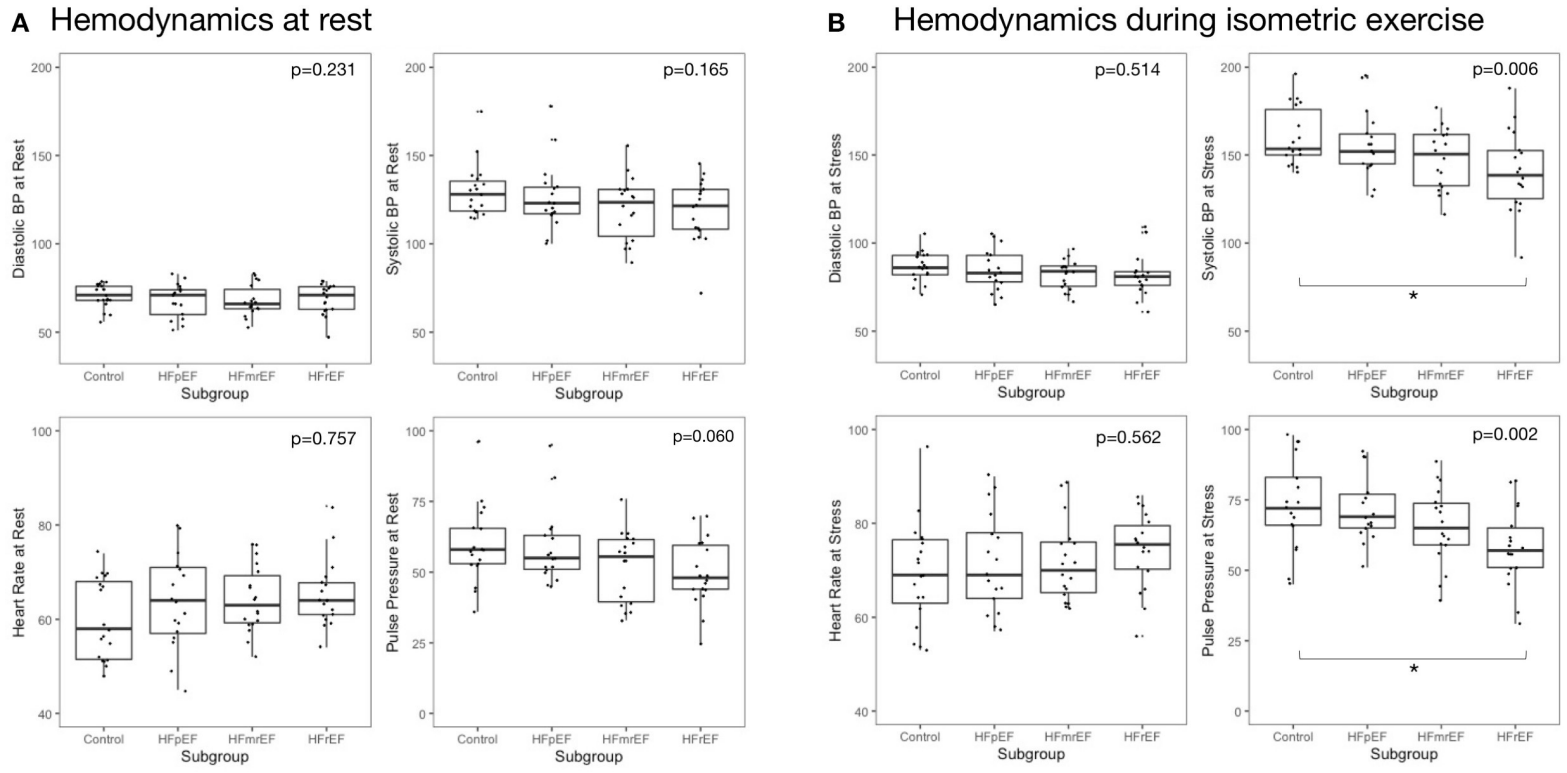
Hemodynamic measurements at rest and during isometric exercise **(A)** at rest and **(B)** during isometric exercise. Reported are *p*-values from analysis of variance. Asterisks indicate significant inter-group difference in Tukey's *post-hoc* test of analysis of variance (*p* < 0.05). BP, blood pressure; EF, ejection fraction; HF, heart failure; HFpEF, HF with preserved EF; HFmrEF, HF with mid-range EF; HFrEF, HF with reduced EF.

Hemodynamic features at rest and during HG are reported in Table 2 and Figure 2. We report change as percentage change to account for subgroup differences at baseline. Numeric differences between rest and HG are reported in Supplementary Table 1. At rest, there were no differences between subgroups in regard of systolic blood pressure (BP), diastolic BP, pulse pressure (PP) or heart rate (HR).

**Table 2:** Hemodynamic characteristics and strain at rest and during isometric exercise.

		**Controls *n* = 19**	**HfpEF *n* = 17**	**HfmrEF *n* = 18**	**HfrEF *n* = 18**	***p-*value**
Heart rate (/min)	Rest	59.9 ± 8.5	63.6 ± 9.8	64.3 ± 7.1	65.2 ± 7.0	0.231
	HG	69.4 ± 10.9*	71.5 ± 10.6*	71.7 ± 8.5*	74.2 ± 8.1*	0.514
	% Change	+16.2 ± 11.8	+12.9 ± 9.4	+11.6 ± 7.1	+14.3 ± 9.7	0.531
Systolic BP (mmHg)	Rest	129.8 ± 14.9	126.5 ± 19.2	119.9 ± 17.8	118.6 ± 17.7	0.165
	HG	163.2 ± 20.2*	156.2 ± 18.8*	147.8 ± 17.3*	140.7 ± 22.8*	0.006
	% Change	+26.3 ± 13.5	+24.8 ± 16.4	+24.1 ± 10.6	+18.9 ± 9.7	0.343
Diastolic BP (mmHg)	Rest	70.5 ± 6.6	67.8 ± 9.7	67.9 ± 8.8	68.8 ± 8.6	0.757
	HG	86.7 ± 8.6*	84.7 ± 11.9*	82.2 ± 8.3*	82.7 ± 13.1*	0.562
	% Change	+23.3 ± 11.5	+25.7 ± 13.7	+22.1 ± 13.3	+20.6 ± 14.7	0.714
Pulse pressure (mmHg)	Rest	59.4 ± 13.4	58.8 ± 13.2	51.9 ± 12.6	49.7 ± 11.9	0.06
	HG	76.5 ± 18.5*	71.5 ± 11.4*	65.6 ± 13.7*	58.1 ± 13.9*	0.002
	% Change	+30.0 ± 22.2	+24.9 ± 25.3	+28.2 ± 18.1	+17.3 ± 11.7	0.238
LV GLS	Rest	−20.1 ± 1.7	−19.1 ± 1.2	−16.0 ± 2.8	−11.4 ± 4.0	<0.001
	HG	−20.2 ± 1.5	−19.0 ± 2.1	−15.6 ± 2.6	−11.0 ± 4.1	<0.001
	% Change	+1.2 ± 5.4	−0.6 ± 8.3	−1.7 ± 10.7	−3.1 ± 19.4	0.746
LV GCS	Rest	−18.7 ± 2.4	−16.9 ± 2.3	−13.0 ± 3.5	−11.2 ± 3.3	<0.001
	HG	−18.4 ± 2.5	−17.2 ± 2.0	−13.1 ± 2.6	−10.6 ± 2.8	<0.001
	% Change	−0.8 ± 11.0	+3.1 ± 11.6	10.8 ± 48.6	−2.4 ± 18.1	0.467

* *Difference between rest and HG significant (p < 0.05), assessed with paired t-test. BP, blood pressure; EF, ejection fraction; GCS, global circumferential strain; GLS, global longitudinal strain; HF, heart failure; HFpEF, HF with preserved EF; HFmrEF, HF with mid-range EF; HFrEF, HF with reduced EF, HG, isometric handgrip; LV, left ventricle*.

In response to HG exercise, systolic and diastolic BP, HR and PP increased in all subgroups. Changes in BP, HR, and PP from rest to HG was not significantly different between subgroups. During HG exercise, we observed a stepwise decrease of systolic BP from controls to subjects with HFpEF, HFmrEF, and HFrEF with 163.2 ± 20.1, 156.2 ± 18.8, 147.8 ±17.3, and 140.7 ± 22.8 mmHg, respectively (*p* = 0.006). A similar pattern was found in PP during HG. Meanwhile, diastolic BP during HG was not different across subgroups.

### Strain at Rest and During Isometric Exercise

**Figure 3:**
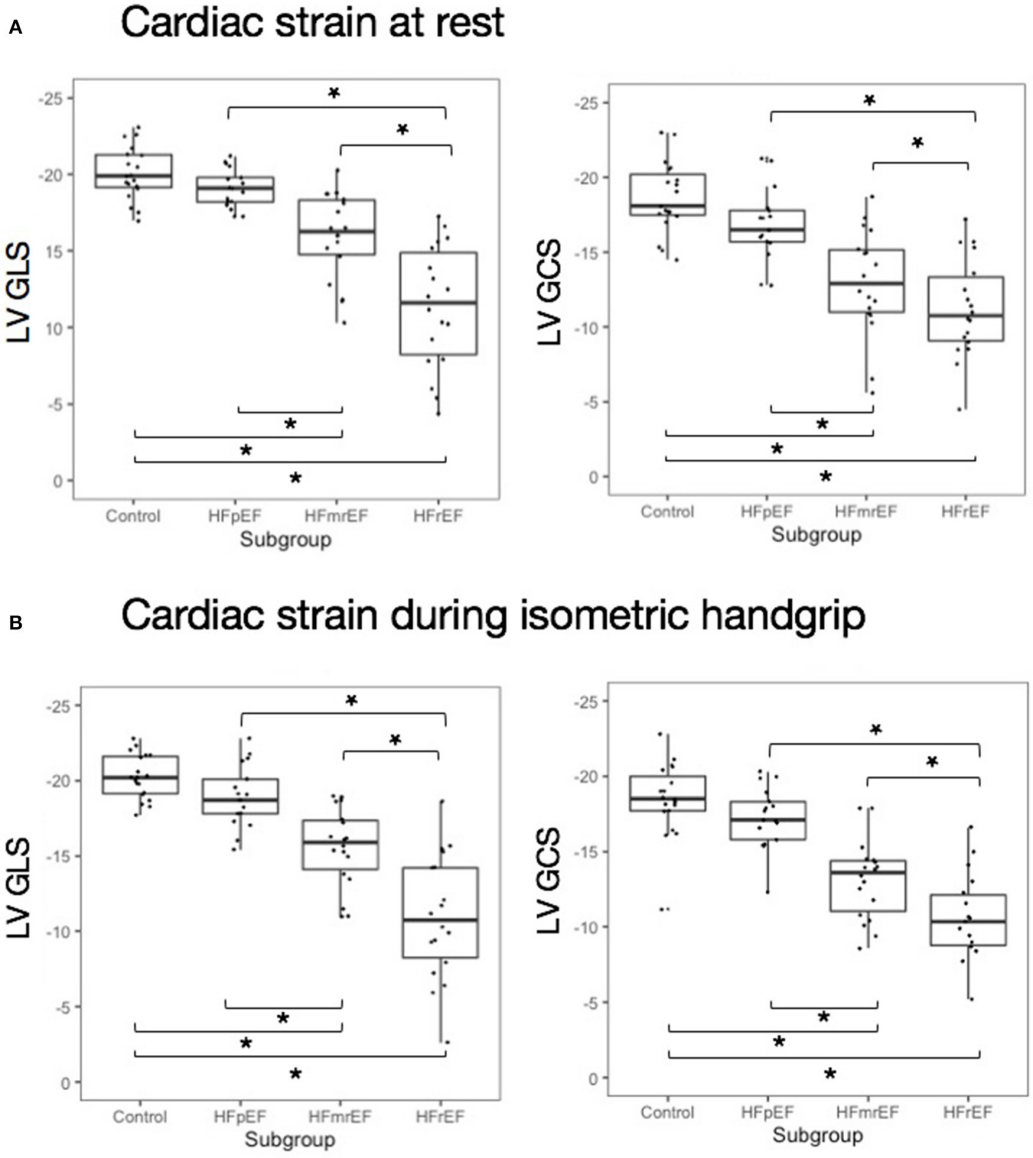
Cardiac strain at rest and during isometric exercise **(A)** at rest and **(B)** during isometric exercise. Asterisks indicate significant inter-group difference in Tukey's *post-hoc* test of analysis of variance (*p* < 0.05). EF, ejection fraction; GCS, global circumferential strain; GLS, global longitudinal strain; HF, heart failure; HFpEF, HF with preserved EF; HFmrEF, HF with mid-range EF; HFrEF, HF with reduced EF; LV, left ventricle.

At rest, LV strain was highest in healthy controls and decreased stepwise with HF category (Table 2, Figure 3). This held true for both LV GLS) and LV GCS. During HG exercise, we found mean LV strain to be largely unchanged. Correspondingly, there was a stepwise decrease with HF category in both LV GLS and LV GCS. A *post-hoc* analysis of subgroup differences in strain and hemodynamic parameters is detailed in Supplementary Table 2.

Mean percentage change between LV GLS at rest and during isometric exercise ranged close to zero with broad standard deviation in all subgroups (Table 2) and was not significantly different between subgroups (+1.2 ± 5.4%, −0.6 ± 8.3%, −1.7 ± 10.7%, and −3.1 ± 19.4%, *p* = 0.746 in controls, HFpEF, HFmrEF, and HFrEF, respectively). LV GLS change and LV GCS change in response to HG exercise were not correlated (*r* = −0.02, *p* = 0.865).

**Figure 4:**
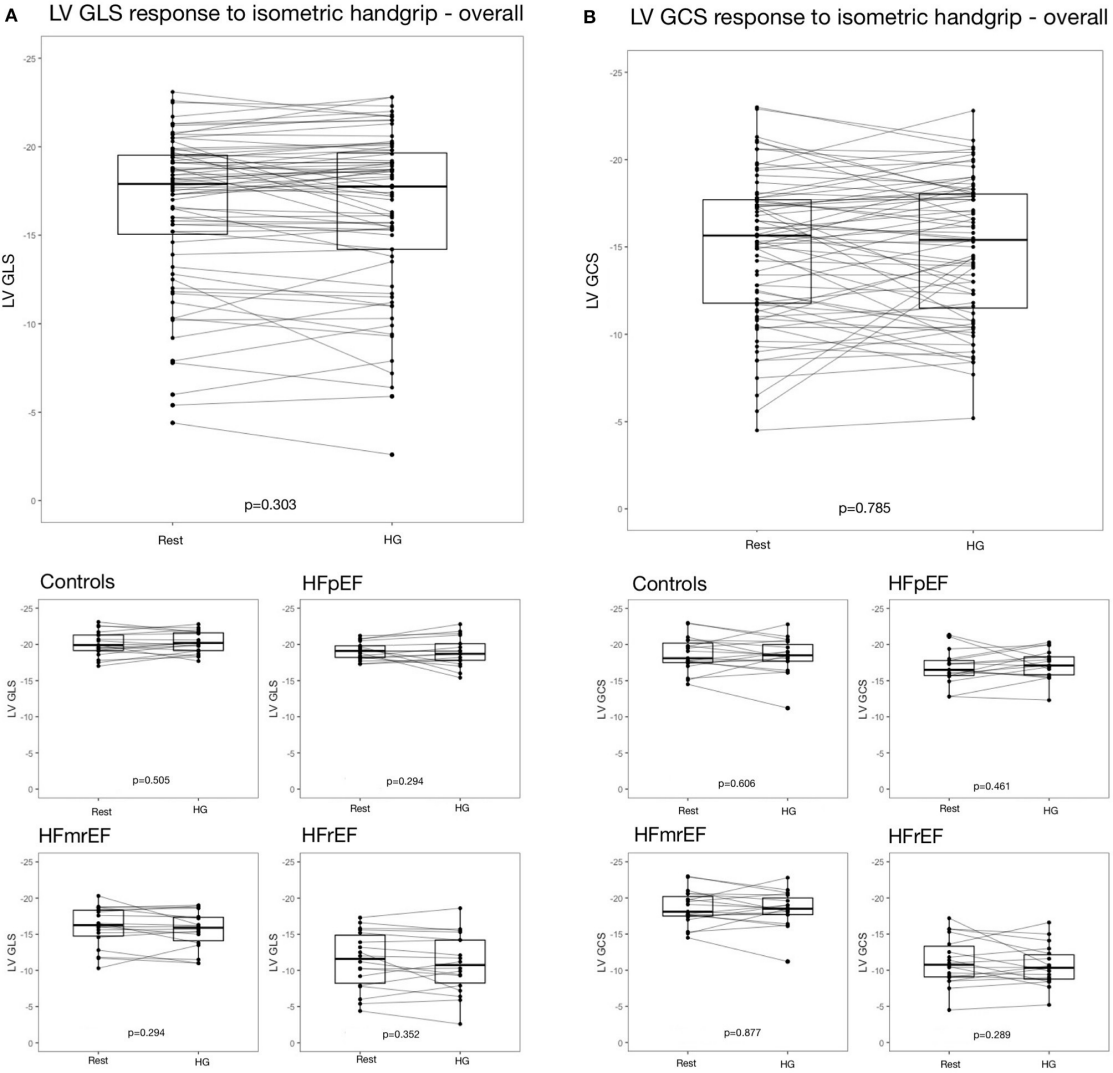
Strain response to isometric exercise **(A)** LV GLS overall and in subgroups, **(B)** LV GCS overall and in subgroups. EF, ejection fraction; GCS, global circumferential strain; GLS, global longitudinal strain; HF, heart failure; HFpEF, HF with preserved EF; HFmrEF, HF with mid-range EF; HFrEF, HF with reduced EF; LV, left ventricle.

On subject level, strain response could be stable, as well as negative or positive (Figure 4). Strain change between ≥-0.5 and ≤ +0.5 was classified as stable as specified above. In controls, LV GLS remained stable in 36.8%, increased in 36.8% and decreased in 26.3% of subjects in response to HG. In HFpEF, HFmrEF and HFrEF patients, similar distributions of LV GLS response to HG were observed (Table 3). The distribution of LV GLS response to HG did not vary significantly between subgroups (*p* = 0.668). There were no differences with regard to baseline characteristics between subjects with increase, decrease and no change of LV GLS in response to HG (Supplementary Table 3). LV GCS response to HG did not vary significantly between subgroups, either (*p* = 0.831).

**Table 3:** Categorization of change in strain during isometric exercise.

		**Controls *n* = 19**	**HfpEF *n* = 17**	**HfmrEF *n* = 18**	**HfrEF *n* = 18**	***p*-value**
LV GLS	Increase–no. (%)	7 (36.8)	6 (35.3)	3 (16.7)	4 (22.2)	0.668
	No change–no. (%)	7 (36.8)	7 (41.2)	9 (50.0)	6 (33.3)	
	Decrease–no. (%)	5 (26.3)	4 (23.5)	6 (33.3)	8 (44.4)	
LV GCS	Increase– no. (%)	7 (36.8)	9 (52.9)	6 (33.3)	7 (38.9)	0.831
	No change– no. (%)	4 (21.1)	4 (23.5)	3 (16.7)	3 (16.7)	
	Decrease–no. (%)	8 (42.1)	4 (23.5)	9 (50.0)	8 (44.4)	

*Increase: Δ LV GLS < −0.5; No change: −0.5 ≤ Δ LV GLS ≤ +0.5; Increase: Δ LV GLS > +0.5; Abbreviations: EF, ejection fraction; GCS, global circumferential strain; GLS, global longitudinal strain; HF, heart failure; HFpEF, HF with preserved EF; HFmrEF, HF with mid-range EF; HFrEF, HF with reduced EF; LV, left ventricle*.

Of note, the range of LV GLS change was narrow in controls (minimum: −11.0%, maximum: +10.0%), but wide in HFrEF (minimum: −42.0%, maximum: +32.0%). This led us to hypothesizing, that the absolute value of strain percentage change, rather than the direction of strain change (i.e., increase or decrease), was associated with presence of HF.

### Absolute Value of Strain Change in Response to Isometric Exercise

Analyzing the absolute i.e., non-negative value of percentage change in strain as a measure of variability rather than increase or decrease in response to HG, we found substantial differences between subgroups (Table 4). In controls, the absolute value of LV GLS change was 4.4 ± 3.2%, in HFpEF it was 5.9 ± 5.7%, in HFmrEF it was 6.8 ± 8.3% and in HFrEF it was 14.1 ± 13.3% (*p* = 0.005). The absolute value of percentage change in LV GCS, again, was lowest in controls (8.6 ± 6.6%) followed by HFpEF (9.8 ± 6.6%), and HFrEF (14.7 ± 10.2%), and highest in HFmrEF (28.3 ± 40.4%, *p* = 0.028).

**Table 4:** Change in strain during isometric exercise.

	**Controls *n* = 19**	**HfpEF *n* = 17**	**HfmrEF *n* = 18**	**HfrEF *n* = 18**	***p-*value**
**LV GLS**					
% change	+1.2 ± 5.4	−0.6 ± 8.3	−1.7 ± 10.7	−3.1 ± 19.4	0.746
Absolute value of % change	4.4 ± 3.2	5.9 ± 5.7	6.8 ± 8.3	14.1 ± 13.3	0.005
**LV GCS**					
% change	−0.8 ± 11.0	+3.1 ± 11.6	+10.8 ± 48.6	−2.4 ± 18.1	0.467
Absolute value of % change	8.6 ± 6.6	9.8 ± 6.6	28.3 ± 40.4	14.7 ± 10.2	0.028

*EF, ejection fraction; GCS, global circumferential strain; GLS, global longitudinal strain; HF, heart failure; HFpEF, HF with preserved EF; HFmrEF, HF with mid-range EF; HFrEF, HF with reduced EF, HG, isometric handgrip; LV, left ventricle*.

**Figure 5:**
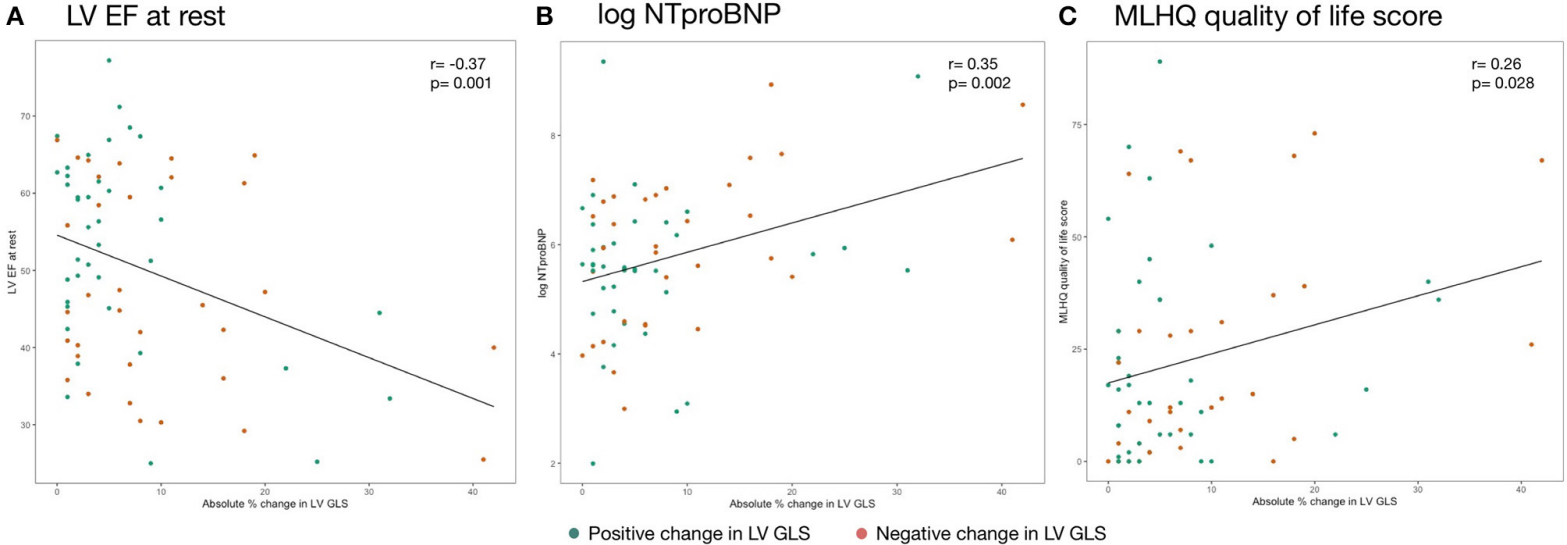
Association of absolute change in strain during isometric exercise and **(A)** LV EF at rest, **(B)** log NTproBNP, and **(C)** MLHQ quality of life score. Reported are Pearson's *r* coefficients. NTproBNP was transformed by the natural logarithm function. EF, ejection fraction; GLS, global longitudinal strain; LV, left ventricle; MLHQ, Minnesota living with heart failure questionnaire; NTproBNP, N-terminal pro-brain natriuretic peptide.

Plotting strain change against various surrogate parameters associated with HF illustrates that the absolute, non-negative value of LV GLS percentage change rather than the direction of this change (i.e., increase or decrease) was associated with disease severity (Figure 5). We further investigated different modes of expressing strain response (i.e., percentage change and the absolute value of percentage change) and their association with clinical, laboratory and imaging parameters. LV GCS change was not correlated with any parameter of HF severity — neither percentage change nor the absolute value of percentage change. Similarly, LV GLS percentage change was not correlated with surrogate parameters of HF severity. The absolute value of LV GLS percentage change, however, was moderately correlated with resting LV EF (*r* = −0.37, *p* = 0.001), NTproBNP (*r* = 0.33, *p* = 0.004), log-transformed NTproBNP (*r* = 0.35, *p* = 0.002), MLHFQ quality of life score (*r* = 0.26, *p* = 0.028), LV end-diastolic volume at rest (*r* = 0.40, *p* = 0.006), and LV end-systolic volume (*r* = 0.43, *p* = 0.001) at rest.

## Discussion

This study investigating cardiac strain in HF patients and controls undergoing cMRI paired with HG yielded the following findings:

1. The response to isometric exercise in LV GLS and GCS is heterogeneous, with increase and decrease in some subjects, and stable strain in others. This pattern was found in controls, as well as in all HF subgroups.
2. In HF patients, the extent of LV GLS change is elevated, regardless of whether strain increases or decreases, when compared to controls. This difference is most pronounced in patients with HFrEF.
3. The absolute value of LV GLS percentage change significantly correlates with surrogate parameters of HF severity.

### Clinical Applications of Strain Assessment

Cardiac strain is a reliable and meaningful tool for detection of myocardial dysfunction in several diseases (2, 7). Multiple studies demonstrated its potential use for early detection of myocardial dysfunction, prognostic stratification and discrimination of different HF entities (8–10). A recent Heart Failure Association consensus recommendation for the diagnosis of HFpEF included impaired GLS into their HFA-PEFF score as a minor criterion (25). Especially in patients with borderline EF, assessment of cardiac strain could facilitate accurate diagnosis of HF, a possibility that future research has to investigate in depth.

The aim of this study, however, was to investigate the feasibility and diagnostic value of cardiac strain measured by fast-SENC in conjunction with HG exercise. Fast-SENC acquisition is rapid, within a single cardiac cycle, making the technique especially helpful for severely ill patients unable to hold breath as in typical cMRI exam (14, 26). It also requires minimal post-processing time to provide accurate and reproducible strain measurements. The SENC images can also be utilized for additional purposes, such as ultra-fast estimation of LV volumes and LV EF (15, 21).

To our best knowledge, our study was the first one to systematically evaluate the combined diagnostic approach of fast-SENC-based LV strain quantification and HG in HF patients and controls.

### Isometric Exercise, Afterload, and Contractility

In spite of only involving a relatively small group of muscles, HG exercise increases cardiac afterload, which has substantial effects on the cardiovascular system (18, 27): Systolic BP, diastolic BP and HR increase markedly which is believed to be due to a circulatory reflex serving to increase perfusion pressure in the contracting muscle groups (28). An early invasive study found that cardiac output (CO) increases during isometric handgrip exercise. However, this increase was mainly driven by a higher heart rate—LV systolic function even decreased slightly (18). A recent meta-analysis of imaging trials investigating the effects of HG on hemodynamic parameters confirmed that HR significantly increases, while SV and CO did not change significantly from rest to HG (29). All these studies support the notion, that the increase in cardiac afterload during HG is predominantly compensated by an increase in HR rather than in systolic myocardial contractility.

Strain has been postulated as the optimal measure of cardiac contraction and multiple studies demonstrated the close relation of strain with other measures of contractility (30–32). Thus, whether strain as a metric of contractility is an adequate measure to characterize the response to increased afterload, remains a question at issue.

### Previous Studies on Strain Response to Increased Afterload

The dependency of myocardial strain on preload as implied by the principles of cardiac mechanics was already established by several early echocardiography studies (33–35). Conversely, the short-term impact of increased afterload on myocardial strain remains controversial.

Fredholm et al. examined 21 patients after cardiac surgery and found no change in strain in response to increased afterload after phenylephrine infusion (36). A study by Stefani et al. analyzed athletes and healthy controls undergoing speckle tracking echocardiography (STE) during HG. The authors found significant changes from baseline longitudinal strain exclusively in the medial to apical myocardial segments of athletes. In controls, no significant change in response to increased afterload was found, whatsoever (37).

On the contrary, a study by Donal et al. of 18 pigs employing open-chest echocardiography during graded aortic banding found a stepwise decrease in longitudinal strain with increasing afterload (38). The authors also found a differences between longitudinal strain, which already deteriorated after moderate increases in afterload (i.e. +10 mmHg) and radial strain, which was preserved during moderate increases in afterload and only deteriorated when afterload was further increased. This study indicates that the different vector components of myocardial strain, i.e. longitudinal, circumferential and radial strain, might react differentially to increased afterload. Of note, quantification of radial strain is technically not possible using fast-SENC (22). A study by Weiner et al. examining 18 healthy subjects undergoing STE found a significant decrease of LV longitudinal strain in response to HG. Simultaneously, parameters of LV twisting decreased significantly (39). Murai et al. observed decreased LV GLS in 41 young and healthy volunteers undergoing a similar STE + HG protocol (23). In addition, they measured ventricular wall stress in order to directly quantify afterload on the myocardium and found that the increase of wall stress and the decrease of strain during HG are inversely correlated. The authors also found that strain rate (SR) was less closely correlated to wall stress, suggesting that SR is less dependent on afterload than strain. All of the previous studies are limited by the shortcomings of hand-held echocardiography, namely angle-dependency of 2D image acquisition and intra-observer variability (7).

### Heterogeneous Strain Response to Isometric Exercise in Controls and HF Patients

Our study expands this limited body of evidence employing a more accurate and reproducible fast-SENC acquisition-based approach to quantify strain and applying isometric HG exercise to increase afterload. Previous studies by our group demonstrated excellent inter-study, inter-observer and intra-observer reproducibility of fast-SENC based LV GLS assessment in both healthy controls and HF patients, providing evidence on the reliability of our strain measurements (15). Since the association of GLS and prognosis in HF is well established, we will focus on LV GLS changes in the following (9). In line with such previous evidence, we also found that the association with indices of HF severity is more pronounced with LV GLS compared to LV GCS.

We found a non-uniform LV GLS response to increased afterload with high variability between subjects. Investigating healthy controls, we found stable LV GLS as well as increase and decrease, with strain changes ranging from a −11.0 to +10.0%. In heart failure patients, strain changes ranged from −42.0 to +32.0%. Our findings imply that assessing strain response to HG based on whether strain increases or decreases might be misleading. Not only in HF patients, but also in healthy subjects, strain response appears to be heterogeneous.

Even though counter-intuitive at first glance, this finding appears to be in line with the preexisting literature: As lined out above, previous evidence on strain response to isometric exercise in non-HF subjects is equivocal with some studies reporting no change in longitudinal strain (36, 37), others reporting decreased GLS during HG (23, 38, 39). Most notably, none of these previous studies elaborated on the heterogeneity of strain response to afterload. Usually, only mean differences are reported. However, in some previous studies figures indicate a mixed response pattern with both increase and decrease of deformation indices present in some subjects (36, 39).

Thus, our observation of a non-uniform LV strain response in controls reconciles contradictory preliminary evidence and explicitly addresses a pattern already implicitly present in previous study reports.

### Increased Absolute Value of Strain Change in HF Patients

In HF patients, LV GLS could be stable as well as increased or decreased in response to isometric exercise. This pattern did not significantly differ between HF patients and controls. However, the extent of strain change irrespective of whether strain increased or decreased was significantly elevated, particularly in HFrEF patients. In addition, the absolute value of LV GLS change was significantly associated with indicators for severity of symptoms. Patients with substantial change in strain—without regard to the direction of change– were more likely to have reduced LV EF, high levels of NTproBNP and to suffer from severe HF symptoms as quantified by MLHFQ. This association with HF severity also raises the question whether extreme strain changes in response to HG might have prognostic implications in HF patients. The significant correlation of the absolute value of LV GLS change and LV end-diastolic and LV end-systolic volumes also establishes a mechanical relationship to cardiac dilation and preload which stipulates further investigation.

The question remaining is what determine decrease or increase of strain in response to HG given that they occur in both healthy and HF subjects. Based on previous studies indicating that increased afterload was compensated by a rise in HR rather than an increase in myocardial contractility, we hypothesized that there might be an association between LV GLS change and with HR change to HG (18, 29). In fact, in an exploratory analysis including only HF patients, we found a significant inverse association between HR change and LV GLS change in response to HG (r = −0.31, *p* = 0.023, Supplemental Figure 1). This supports the notion that strain change and HR change are inversely related and excessive increase in LV GLS might be an expression of inability to adjust HR in response to isometric exercise. However, this association dissipated when including controls into this exploratory analysis (*r* = −0.20, *p* = 0.095).

### Implications for Future Research Into Strain Response to Increased Afterload

The potential association of strain change and HR change again points toward SR, i.e. the temporal derivative of strain, as a promising measure for future studies into the effects of HG on myocardial contractility. SR depends on both strain and the length of the cardiac cycle (40). During HG exercise, HR physiologically increases leading to a shortening of myocardial contraction time per heartbeat. With strain decreasing while the cardiac cycle is shortening in response to increased afterload, SR which is a function of strain and contraction time might stay relatively stable. Murai et al. in fact demonstrated that SR is less dependent on afterload than strain in healthy subjects (23). Thus, significant changes in SR during HG could potentially reflect dysbalance regarding response of strain and HR to increased afterload in HF patients. However, due to software limitations we were not able to quantify SR in this study.

Furthermore, it is important to bear in mind that the LV is not operating mechanically in isolation. Both the right ventricle (RV) and the left atrium (LA) have been identified to play important roles in exercise hemodynamics (41, 42). Thus, further investigation into LA strain, RV strain and their relation to LV strain in exercise settings with increased afterload are necessary in order to fully understand cardiac deformation mechanics during HG.

Even though our findings elucidate cardiac adaption mechanisms in response to acute increase in afterload, our study suggests that the diagnostic utility of strain assessment in conjunction with HG is limited. With heterogeneous response patterns and dependency on heart rate variability and presumably other factors not yet fully understood, assessment of strain response to isometric exercise does not appear to provide substantial additional diagnostic value on top of strain assessment during physical rest. Other stress testing modalities such as pharmacological stress induction and dynamic exercise testing have more drastic effects on HR and stroke volume and might be better suited for diagnostic purposes (29).

### Limitations

Several limitations of this study have to be addressed. We cannot rule out the possibility of confounding by unmeasured variables. While including more patients than previous studies investigating the impact of increased afterload on strain, our sample size was still relatively small. We had to exclude patients with implanted ICD and Pacemakers due to MRI contraindication. This limits the generalizability of our study to the general HF population, especially in patients with HFrEF. Concomitant medication, namely BB, might have influenced the hemodynamic response to isometric exercise, particularly regarding the physiological increase in HR. Similarly, left bundle branch block in particular is known to compromise cardiac adaption to increased afterload and different prevalence of LBBB within different subgroups might have impacted our findings (43). Also, we cannot rule out the possibility that ischemia-related motion abnormalities influenced our findings. Besides, HG exercise testing is prone to measurement errors due to lack of cooperation or Valsalva-maneuver during handgrip leading to elevated intrathoracic pressures. Diligent patient instruction and supervision during exercise by trained personnel was implemented to prevent such errors. However, our findings should only be considered hypothesis-generating.

## Conclusion

In conclusion, we found that the strain response to isometric exercise quantified by fast-SENC is heterogeneous: LV GLS and GCS are stable in some patients, but decrease or increase in others, with no significant differences between controls and HF subgroups. However, the absolute value of strain change during isometric exercise—rather than increase or decrease—is elevated in HF patients and associated with measures of HF severity. Our observations indicate that the applicability of strain assessment in conjunction with HG for diagnostic purposes in HF seems to be limited.

## Data Availability Statement

The datasets presented in this article are not readily available because the informed consent given by study participants allows for data sharing only with parties which are explicitly mentioned in the consent form. Requests to access the datasets should be directed to Sebastian Kelle, kelle@dhzb.de.

## Ethics Statement

The studies involving human participants were reviewed and approved by Ethikausschuss 4 am Campus Benjamin Franklin, Charité Universitätsmedizin Berlin. The patients/participants provided their written informed consent to participate in this study.

## Author Contributions

MB, DH, H-DD, and SK conceived and designed the study. MB, DH, LM, MN, AD, and KR acquired clinical data. VZ, SZ, TL, and SK acquired and analyzed imaging data. MB executed the statistical analysis and drafted the manuscript. MK, TK, ET, BP, FE, H-DD, and SK revised and amended critical parts of the manuscript. All authors contributed to the interpretation of the data and approved the final version of this manuscript.

## Conflict of Interest

SK reports grants and other support by the DZHK (German Center for Cardiovascular Research), Partner Site Berlin, Philips Healthcare, BioVentrix, Berlin-Chemie, Merck/Bayer, Novartis, Astra Zeneca, Siemens and Myocardial Solutions outside of the submitted work. SK was also on the advisory board for Merck/Bayer, BioVentrix, and Myocardial Solutions. BP has provided steering committee and advisory board services for Bayer Healthcare and MSD; and has received steering committee and advisory board/speaker honoraria from Novartis. The remaining authors declare that the research was conducted in the absence of any commercial or financial relationships that could be construed as a potential conflict of interest.
