# Supplementary material for: Variability of Myocardial Strain During Isometric Exercise in Subjects With and Without Heart Failure
Source: Front Cardiovasc Med. 2020 Jun 30;7:111. doi: 10.3389/fcvm.2020.00111 (PMC7344153; doi:10.3389/fcvm.2020.00111)
Supplement: Supplementary file 1 [file Data_Sheet_1.pdf]

Supplementary Material

Variability of Myocardial Strain During Isometric Exercise in Subjects With and Without Heart Failure

Moritz Blum[1], Djawid Hashemi[1,2], Laura Astrid Motzkus[1], Marthe Neye[1], Aleksandar Dordevic[1], Victoria Zieschang[3], Seyedeh Mahsa Zamani[3], Tomas Lapinskas[3,4], Kilian Runte[5,6], Marcus Kelm[5,6], Titus Kühne[2,5,6], Elvis Tahirovic[1,2], Frank Edelmann[1,2], Burkert Pieske[1,2,3], Hans-Dirk Düngen[1,2], Sebastian Kelle[1,2,3*]

[1] Department of Internal Medicine/Cardiology, Charité–Universitätsmedizin Berlin, Germany

[2] DZHK (German Center for Cardiovascular Research), partner site Berlin, Germany

[3] Department of Internal Medicine/Cardiology, German Heart Center Berlin, Germany

[4] Department of Cardiology, Medical Academy, Lithuanian University of Health Sciences, Kaunas, Lithuania.

[5] Department of Congenital Heart Disease, German Heart Center Berlin, Germany

[6] Institute for Imaging Science and Computational Modelling in Cardiovascular Medicine, Charité–Universitätsmedizin Berlin, Germany

*** Correspondence:**
Sebastian Kelle, MD, PhD
kelle@dhzb.de; sebastian.kelle@charite.de

1 Supplementary Figures and Tables

1.1 Supplementary Figures

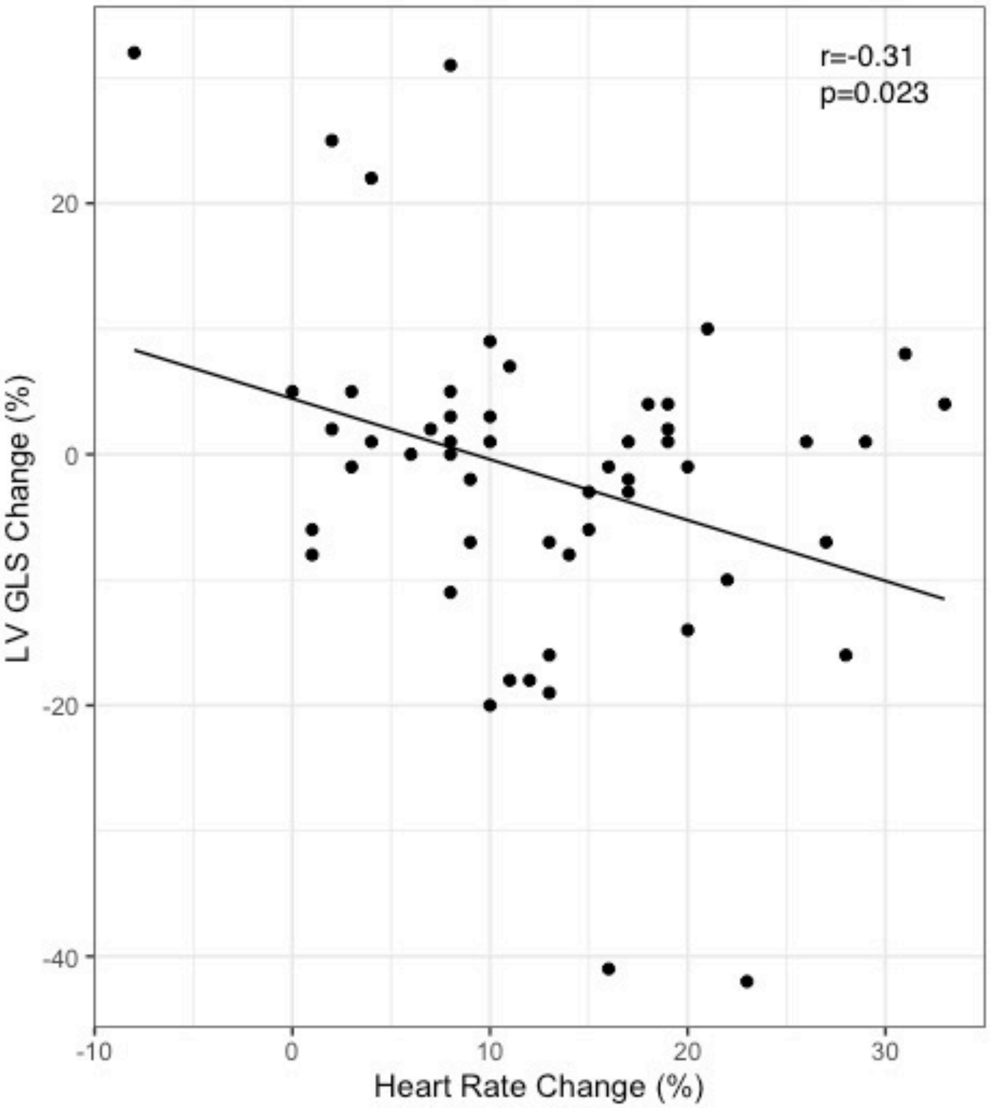

Supplementary Figure 1. Association of heart rate change and LV GLS change in response to isometric exercise. LV GLS, left ventricular global longitudinal strain. Only subjects with heart failure were included in this exploratory analysis.

1.2 Supplementary Tables

Supplementary Table 1. Numeric difference between rest and isometric exercise					
	**Controls** n=19	**HFpEF** n=17	**HFmrEF** n=18	**HFrEF** n=18	**p-value**
Δ Heart rate (/min)	+9.5 ± 6.5	+7.9 ± 5.1	+7.4 ± 4.6	+9.1 ± 6.1	0.663
Δ Systolic BP (mmHg)	+33. ± 17.8	+29.6 ± 17.4	+27.9 ± 10.6	+22.2 ± 11.7	0.148
Δ Diastolic BP (mmHg)	+16.2 ± 7.5	+16.9 ± 8.2	+14.3 ± 7.9	+13.8 ± 10.6	0.658
Δ Pulse Pressure (mmHg)	+17.2 ± 13.3	+12.7 ± 12.4	+13.6 ± 7.0	+8.3 ± 5.8	0.082
Δ LV GLS	-0.2 ± 1.0	+0.1 ± 1.6	+0.4 ± 1.5	+0.4 ± 1.7	0.626
Δ LV GCS	+0.2 ± 1.9	-0.4 ± 2.1	-0.1 ± 3.8	+0.6 ± 2.3	0.702
Abbreviations: BP, blood pressure; EF, ejection fraction; GCS, global circumferential strain; GLS, global longitudinal strain; HF, heart failure; HFpEF, HF with preserved EF; HFmrEF, HF with mid-range EF; HFrEF, HF with reduced EF, isoHG, isometric handgrip; LV, left ventricle.					

Supplementary Table 2. Post-hoc subgroup comparisons		
**Variable**	**Comparison**	**P-value**
Systolic BP during IsoHG	HFpEF-Control	0.716
	HFmrEF-Control	0.096
	HFrEF-Control	0.005
	HFmrEF-HFpEF	0.599
	HFrEF-HFpEF	0.109
	HFrEF-HFmrEF	0.713
Pulse Pressure during IsoHG	HFpEF-Control	0.733
	HFmrEF-Control	0.116
	HFrEF-Control	0.001
	HFmrEF-HFpEF	0.636
	HFrEF-HFpEF	0.043
	HFrEF-HFmrEF	0.426
LV GLS at Rest	HFpEF-Control	0.683
	HFmrEF-Control	<0.001
	HFrEF-Control	<0.001
	HFmrEF-HFpEF	0.006
	HFrEF-HFpEF	<0.001
	HFrEF-HFmrEF	<0.001
LV GLS during IsoHG	HFpEF-Control	0.510
	HFmrEF-Control	<0.001
	HFrEF-Control	<0.001
	HFmrEF-HFpEF	0.003
	HFrEF-HFpEF	<0.001
	HFrEF-HFmrEF	<0.001
LV GCS at Rest	HFpEF-Control	0.267
	HFmrEF-Control	<0.001
	HFrEF-Control	<0.001
	HFmrEF-HFpEF	0.001
	HFrEF-HFpEF	<0.001
	HFrEF-HFmrEF	0.290
LV GCS during IsoHG	HFpEF-Control	0.487
	HFmrEF-Control	<0.001
	HFrEF-Control	<0.001
	HFmrEF-HFpEF	<0.001
	HFrEF-HFpEF	<0.001
	HFrEF-HFmrEF	0.020

Hemodynamic and strain variables which differed significantly between subgroups when assessed with analysis of variance were included in this post-hoc analysis using Tukey's test. Abbreviations: BP, blood pressure; GCS, global circumferential strain; GLS, global longitudinal strain; IsoHG, isometric handgrip; LV, left ventricle.

Supplementary Table 3. Characteristics of patients with increase, no change and decrease of LV GLS in response to isometric exercise					
		**Increase** n=20	**No change** n=29	**Decrease** n=23	**p-value**
**Subgroup**					0.668
**Control** – no. (%)		7 (35.0)	7 (24.1)	5 (21.7)	
**HFpEF** – no. (%)		6 (30.0)	7 (24.1)	4 (17.4)	
**HFmrEF** – no. (%)		3 (15.0)	9 (31.0)	6 (26.1)	
**HFrEF** – no. (%)		4 (20.0)	6 (20.7)	8 (34.8)	
**Female Sex** – no. (%)		8 (40.0)	10 (34.5)	8 (34.8)	
**Age** – years		69.80 ± 8.28	71.00 ± 10.83	68.21 ± 10.52	0.738
**BMI** – kg/m^2		27.66 ± 4.37	26.55 ± 4.19	26.94 ± 3.27	0.632
**CAD** – no. (%)		11 (55.0)	18 (62.1)	10 (43.5)	0.408
**Hypertension** – no. (%)		15 (75.0)	19 (65.5)	17 (73.9)	0.715
**Previous MI** – no. (%)		9 (45.0)	13 (44.8)	7 (30.4)	0.506
**Previous PCI** – no. (%)		10 (50.0)	15 (51.7)	10 (43.5)	0.831
**Diabetes mellitus** – no. (%)		6 (30.0)	7 (24.1)	2 (8.7)	0.195
**LBBB on ECG** – no. (%)		1 (5.0)	1 (3.4)	1 (4.3)	0.946
**Ever Smoked** – no. (%)		10 (50.0)	17 (58.6)	13 (56.5)	0.832
**Packyears – years**		15.79 ± 34.99	19.16 ± 41.97	16.91 ± 20.94	0.945
**NYHA Class**	**II** – no. (%)	10 (66.7)	16 (69.6)	13 (68.4)	0.999
	**III** – no. (%)	4 (26.7)	6 (26.1)	5 (26.3)	
**Leg Edema** – no. (%)		11 (55.0)	17 (58.6)	13 (56.5)	0.757
**6 min walk distance** – m		410.70 ± 132.67	411.32 ± 119.76	462.57 ± 130.05	0.284
**MLHFQ QOL Score**		24.15 ± 24.20	17.69 ± 19.11	27.13 ± 25.15	0.31
**Beta-Blocker** – no. (%)		14 (70.0)	19 (65.5)	15 (65.2)	0.933
**ACE-Inhibitor** - no. (%)		4 (20.0)	8 (27.6)	9 (39.1)	0.376
**ARB** – no. (%)		10 (50.0)	11 (37.9)	10 (43.5)	0.703
**MRA** – no. (%)		5 (25.0)	5 (17.2)	8 (34.8)	0.349
**ARNI** – no. (%)		1 (5.0)	1 (3.4)	3 (13.0)	0.37
**Statin** – no. (%)		12 (60.0)	16 (55.2)	9 (39.1)	0.342
**Loop Diuretic** – no. (%)		4 (20.0)	7 (24.1)	5 (21.7)	0.941
**HCT** – no. (%)		3 (15.0)	5 (17.2)	3 (13.0)	0.916
**Hb** – g/dl		13.72 ± 1.79	13.87 ± 1.15	14.09 ± 1.09	0.665
**RBC** – /pl		4.65 ± 0.50	4.69 ± 0.47	4.60 ± 0.48	0.784
**WBC** – /nl		7.77 ± 2.10	7.25 ± 2.54	7.61 ± 2.33	0.73
**Platelets** – /nl		276.50 ± 58.93	245.76 ± 63.14	236.78 ± 81.54	0.148
**Hematocrit**		0.41 ± 0.05	0.41 ± 0.03	0.41 ± 0.03	0.989
**Cholesterol** – mg/dl		163.65 ± 37.23	174.18 ± 46.76	177.39 ± 40.74	0.546
**LDL** – mg/dl		101.80 ± 32.65	106.36 ± 45.57	107.65 ± 35.91	0.878
**HDL** – mg/dl		52.50 ± 17.63	55.21 ± 22.08	57.78 ± 18.22	0.682

**Triglycerides** – mg/dl	143.95 ± 103.46	129.36 ± 93.37	158.52 ± 102.61	0.582
**HbA1c** – %	5.74 ± 0.77	5.83 ± 0.76	5.59 ± 0.58	0.491
**NTproBNP** – ng/l	747.60 ± 1909.24	782.74 ± 2083.60	1089.57 ± 1802.21	0.808
**Hs TroponinT**– ng/l	17.90 ± 17.69	16.36 ± 17.14	13.55 ± 9.98	0.648
**CRP** – mg/l	1.96 ± 1.82	2.40 ± 3.74	1.70 ± 1.44	0.64
**LVEF** – %	54.50 ± 13.78	49.98 ± 11.47	47.49 ± 13.47	0.202
**LV EDV** – ml	165.07 ± 63.44	177.56 ± 62.06	193.70 ± 68.92	0.351
**LV ESV** – ml	80.29 ± 54.96	94.59 ± 53.74	108.06 ± 63.57	0.292
**LV SV** – ml	83.36 ± 17.36	83.10 ± 16.00	85.36 ± 18.75	0.885

Increase: Δ LV GLS < -0.5; No change: -0.5 ≤ Δ LV GLS ≤ +0.5; Increase: Δ LV GLS > +0.5; Non-standard abbreviations: ARB, angiotensin receptor blocker; ARNI, angiotensin receptor blocker – neprilysin inhibitor; BP, blood pressure EDV, end-diastolic volume; EF, ejection fraction; ECG, electrocardiogram; ESV, end-systolic volume; GCS, global circumferential strain; GLS, global longitudinal strain; HF, heart failure; HFpEF, HF with preserved EF; HFmrEF, HF with mid-range EF; HFrEF, HF with reduced EF; LBBB, left bundle branch block; MI, myocardial infarction; MLHFQ, Minnesota living with heart failure questionnaire; MRA, mineralocorticoid receptor antagonist, PCI, percutaneous coronary intervention; QOL, quality of life; RBC, red blood cells; WBC, white blood cells.
